# Role of *t-PA* and *PAI-1* variants in temporal lobe epilepsy in Chinese Han population

**DOI:** 10.1186/s12883-019-1239-0

**Published:** 2019-01-22

**Authors:** Wenxiu Han, Pei Jiang, Yujin Guo, Pengfei Xu, Ruili Dang, Gongying Li, Xin He, Dehua Liao, Genquan Yan

**Affiliations:** 1 Institute of Clinical Pharmacy & Pharmacology, Jining First People’s Hospital, Jining Medical University, Jining, 272000 China; 2 Department of Mental Health, Jining Medical University, Jining, 272000 China; 3 Department of Pharmacy, Second Xiangya Hospital, Central South University, Changsha, 410010 China; 4 Department of Pharmacy, Hunan Cancer Hospital, Central South University, Changsha, 410011 China; 5 Department of Pharmacy, Shandong Provincial Hospital affiliated to Shandong University, Jinan, 250100 China

**Keywords:** *t-PA*; *PAI-1*, Temporal lobe epilepsy, Polymorphism

## Abstract

**Background:**

Epilepsy is one of the most common chronic disabling neurologic diseases. The purpose of our study was to investigate whether there is an association between *t-PA* (tissue plasminogen activator, rs2020918 and rs4646972), *PAI-1* (plasminogen activator inhibitor 1, rs1799768) polymorphisms and susceptibility to temporal lobe epilepsy (TLE) in Chinese Han population.

**Method:**

One hundred and twenty-one cases of patients who were diagnosed as TLE and 146 normal controls were enrolled and the genotypes of *t-PA* and *PAI-1* were detected by polymerase chain reaction-ligase detection reaction (PCR-LDR) method after the genomic DNA being extracted from peripheral blood.

**Result:**

There were significant differences for the genotypic frequencies at the two polymorphic sites in *t-PA* gene between TLE patients and controls (*P* = 0.019; *P* = 0.001). Furthermore, the frequency of rs2020918 (C **>** T) with T (CT + TT) and rs4646972 (311 bp insertion/−) with 311 bp deletion (311 bp/− + −/−) was significantly higher among TLE patients relative to controls respectively (*P* = 0.006; *P* = 0.001). However, no significant difference in genotypic and allelic frequency was found at the polymorphic site in *PAI-1* gene between TLE patients and controls (*P* = 0.735).

**Conclusion:**

We reported for the first time to our knowledge the significant role of the two SNPs in *t-PA* gene (rs2020918 and rs4646972) in developing susceptibility to TLE in Chinese Han population.

## Background

Epilepsy is a chronic disorder characterized by recurrent seizures, which affects 8 in 1000 children and children are more frequently affected than adults (children to adult ratio is 2:1) [1–3]. As one of the most common forms of epilepsy, partial seizure is initially focused in just one part of the brain. Among them, more than 1/3 of patients manifested as temporal lobe epilepsy (TLE) [4]. Genetic factors together with environmental factors may contribute to the occurrence, development and prognosis of TLE. Most of the clinical TLE cases are sporadic and may be affected by a combination of multiple pathogenic genes. Emerging evidence suggests that several genetic defects in Interleukin-1β *(IL-1β)* [5], Interleukin − 1 receptor antibody *(IL-1 RA)* [6], γ-Aminobutyric acid B receptor 1*(GABBR1)* [7], prodynorphin *(PYDN)* [8], ApolipoproteinE *(ApoE)* [9] and prion protein *(PRNP)* [10] have been associated with TLE.

In addition to the well-known ion channels and signaling pathways involved in TLE etiology, tissue plasminogen activator (t-PA) is responsible for the activation of plasminogen to plasmin, which then degrades extracellular matrix (ECM) components, promoting synaptic plasticity and influencing neurite sprouting and extension [11]. Abnormalities including the nature and quantity in *t-PA* may be involved in the synaptic plasticity alterations and abnormal neurite extension, leading to the susceptibility of epilepsy. As a member of the serpin family proteinase inhibitors, plasminogen activator inhibitors (PAI-1) can specifically bind to t-PA and terminate the t-PA enzymatic activity in the extracellular space [12], which also may be involved in epileptic seizures. Studies have shown that t-PA is highly enriched in all kinds of types of neurons in the human central nervous system (CNS), including neocortex, pyramidal neurons, and hippocampus, and thus is involved in several physiological and pathological processes in the CNS, such as learning and memory, anxiety, epilepsy, stroke, Alzheimer’s disease, and spinal cord injuries [13, 14]. As expected, elevated *t-PA* mRNA levels were found in the hippocampus [15] and entorhinal cortex [16] of animal models of epilepsy induced by electricity. Consistent with the findings above, elevated *t-PA* mRNA levels were also detected in epilepsy patients [17], which identified a role for *t-PA* in the mechanisms of underlying seizure activity. In addition, PAI-1 becomes a key factor in epileptic seizures due to its high affinity for t-PA. Increased levels of *PAI-1* mRNA have also been observed in human TLE with hippocampal sclerosis and focal cortical dysplasia [17]. Based on the above background, we aimed to investigate the association between *t-PA* (rs2020918, rs4646972), *PAI-1* SNPs (rs1799768) and susceptibility to TLE in Chinese Han population.

## Methods

### Study population

A total of 121 (70 boys and 51 girls, sex ratio 1.37:1.0, mean age at seizure onset 6.4 ± 3.27 y) cases of TLE patients were enrolled in our study. All the patients were recruited at the outpatient clinic of the Second Xiangya Hospital in Hunan Province. The TLE was diagnosed by comprehensive evaluation of characteristic partial seizure symptoms. Criteria for the diagnosis and exclusion of the TLE patients have been described previously [18, 19]. Briefly, since electroencephalography (EEG) and magnetic resonance imaging (MRI) criteria are considered to be reliable interictal indicators of TLE, the diagnosis of TLE was mainly based on typical temporal auras or ictal and interictal EEG discharges over the temporal lobes in the presence of focal spikes or sharp waves followed by slow waves. Patients with any mass lesion such as tumor, cortical dysgenesis, vascular lesion, malformation, or posttraumatic scars detected by MRI were excluded. None had mental retardation, psychiatric difficulties, and early psychiatric manifestations. We also enrolled 146 healthy control subjects (76 males and 70 females) without a history of seizures, related family histories or inherited CNS diseases. Our study was approved by the medical ethics committee of the Second Xiangya Hospital of Central South University. Written informed consent was signed by each participant or their parents/legal guardians before they participated in the study.

### Genetic studies

Genomic DNA was extracted from peripheral blood using TIANamp Blood DNA Kit (TIANGEN, China), according to the manufacturer’s recommendations. Polymerase chain reaction-ligase detection reaction (PCR-LDR) method was used to identify the genotypes. All primers for both PCR and LDR reaction were designed by online software Primer 3 (shown in Table 1). These PCR products and the LDR probes were then subjected to a multiplex ligase detection reaction, with a DNA sequencer used to detect the products. In addition, not less than 10% of DNA samples were randomly selected and genotyped again for the purpose of quality control of the genotyping.

**Table 1:** Primers of Target Gene Used in the PCR

SNP	Ancestor allele	Primer sequence	Product size (bp)
rs2020918	C	5’-CAGATAATTCCTTCTGACCC-3′(forward) 5’-TGAATAGGGCTTTGGCCGCT-3′(reverse)	117
rs4646972	not available	5’-AGTTAAGGGTCCTGGCCTGT −3′(forward) 5′- TCATCTTGACCTTGCAGCAC -3′ (reverse)	219
rs1799768	T	5’-CAACCTCAGCCAGACAAGGT-3′ (forward) 5’-CAGCCACGTGATTGTCTAGG-3′ (reverse)	192

### Statistical analysis

The Hardy–Weinberg Equilibrium test was performed for every single-nucleotide polymorphism in controls. The Person χ^2^ test was used to compare the statistical differences in genotype distributions and allele frequencies between TLE patients and controls. Moreover, Bonferroni adjustment was performed to correct for multiple comparisons. The odds ratio (OR) and 95% confidence intervals (95%CI) were also calculated. A *P* value < 0.05 was considered as significant. All analyses were conducted using statistical software package SPSS 17.0 (SPSS Inc., Chicago, IL, USA).

## Results

Genotype distribution in control groups was consistent with Hardy–Weinberg Equilibrium according to chi-square test (rs2020918: χ^2^ = 0.971, *P* = 0.324; rs4646972: χ^2^ = 3.699, *P* = 0.054; rs1799768: χ^2^ = 0.138, *P* = 0.710).

### *t-PA* polymorphisms

The results of genotypic and allelic frequencies in rs2020918 and rs4646972 of *t-PA* gene were summarized in Tables 2 and 3. There was a significant difference in the frequency of the *t-PA* polymorphism rs2020918 (C > T) (χ^2^ = 7.939, *P* = 0.019) between TLE patients and healthy controls; however, the significance of this result was not confirmed after the strict Bonferroni adjustment (*P* = 0.057). When subdividing these samples into CC and CT+ TT groups, an obvious difference between cases and controls was found (χ^2^ = 7.670, *P* = 0.006, OR = 2.008, 95% CI = 1.223–3.298). Moreover, the T allele showed a significant association with TLE patients group (χ^2^ = 8.098, *P* = 0.004, OR = 1.789, 95% CI = 1.195–2.678). Similarly, for rs4646972, the genotypic and allelic distributions between two groups were statistically different after the strict Bonferroni adjustment (χ^2^ = 13.366, *P* = 0.003 by genotype; χ^2^ = 15.630, *P* = 0.000, OR = 2.007, 95% CI = 1.418–2.840 by allele). The frequency of genotype with 311 bp deletion (311 bp/− + −/−) was significantly more frequent among the TLE patients and the controls (χ^2^ = 10.517, *P* = 0.001, OR = 0.422, 95% CI = 0.250–0.715).

**Table 2:** Genotypic distribution of the *t-PA* and *PAI-1* gene between TLE patients and controls

Gene	SNP	Genotype	Cases (242 alleles across 121 subjects) n (% of population)	Controls (292 alleles across 146 subjects) n (% of population)	OR(95%CI)	χ^2^	*p*-value^a^	*p*-value^b^
*t-PA*	rs2020918	CC	61(50.4)	98(67.1)				
		CT	49(40.5)	41(28.1)				
		TT	11(9.1)	7(4.8)		7.939	0.019^a^	0.057^b^
		CT + TT	60(49.6)	48(32.9)	2.008(1.223–3.298)	7.670	0.006	
	rs4646972	311 bp/311 bp	30(24.8)	64(43.8)				
		311 bp/−	52(43.0)	57(39.1)				
		−/−	39(32.2)	25(17.1)		13.366	0.001^a^	0.003^b^
		311 bp/− + −/−	91(75.2)	82(56.2)	0.422(0.250–0.715)	10.517	0.001	
*PAI-1*	rs1799768	GG	20(16.5)	27(18.5)				
		−/G	63(52.1)	69(47.3)				
		−/−	38(31.4)	50(34.2)		0.616	0.735	

CT + TT: the sum of CT and TT genotype

311 bp/− + −/−: the sum of 311 bp/− and −/− genotype

^a^*p*-value without adjustment

^b^*p*-value after Bonferroni adjustment for multiple comparisons

**Table 3:** Allelic distribution of the *t-PA* and *PAI-1* gene between TLE patients and controls

Gene	SNP	Allele	Cases (242 alleles across 121 subjects) n (% of population)	Controls (292 alleles across 146 subjects) n (% of population)	OR(95%CI)	χ^2^	*p*-value
*t-PA*	rs2020918	C	171(70.7)	237(81.2)			
		T	71(29.3)	55(18.9)	1.789(1.195–2.678)	8.098	0.004
	rs4646972	311 bp	112(46.3)	185(63.4)			
		–	130(53.7)	107(36.6)	2.007(1.418–2.840)	15.630	0.000
*PAI-1*	rs1799768	–	139(57.4)	169(57.9)			
		G	103(42.6)	123(42.1)	1.018(0.721–1.437)	0.01	0.919

### *PAI-1* polymorphism

The results of genotypic and allelic frequencies in rs1799768 of *PAI-1* gene were presented in Tables 2 and 3. No significant differences were observed at the polymorphic sites between the two groups in terms of genotypic distributions (χ^2^ = 0.616, *P* = 0.735), nor for allelic frequencies (χ^2^ = 0.01, *P* = 0.919, OR = 1.018, 95% CI = 0.721–1.437).

## Discussion

The human gene *t-PA (PLAT)* spanning more than 30 kb is located on chromosome 8p11.21 and consists of 14 exons, encoding tissue plasminogen activator [20]. As a key protease of the fibrinolytic system, t-PA is localized broad regions including cortex, cerebellum, amygdala, and hippocampus, and thus has essential physiological and pathological functions in CNS. As the specific inhibitor of t-PA in plasma, plasminogen activator inhibitor type-1 (PAI-1) is encoded by *PAI-1* (also known as *SERPINE1*) gene, which is located at 7q21.1 and consists of 9 exons and 8 introns [21]. Genetic variations have been shown to play a vital role in regulating the level of t-PA and the three mostly studied SNPs (rs2020918, rs4646972 in *t-PA* and rs1799768 in *PAI-1*) have been associated with the occurrence of a variety of diseases [20, 22, 23]. Specifically, the C-T transition at − 7351 (rs2020918, -7351C > T) within the *t-PA* enhancer can cause a potential weakening of the enhancement, which may affect the regulation of *t-PA* expression. Likewise, an Insertion/Deletion of 311 bp sequence polymorphism (rs4646972) identified in the 8th intron of the *t-PA* gene may affect the splicing of mRNA, resulting in the alterations of t-PA function. Interestingly, both of these two SNPs above have been shown to be strongly correlated to t-PA release [22]. As accumulated evidences identified its pivotal role in the mature of pro-neurotrophins [24, 25] and activation of receptors [26], altered t-PA level may be associated with a series of neurological diseases such as epilepsy [27]. In addition, another single guanine nucleotide Insertion/Deletion polymorphism (−/G) is located in the promoter region of the *PAI-1* gene, which was claimed to influence the expression of *PAI-1* [22]. Although previous data showed that these SNPs were not only associated with multiple thrombotic disorders, such as strokes [20, 22, 28], myocardial infarction [29–31], but also with the severity of bacterial infections, such as meningitis [32, 33], it remains blank concerning the relationship between *t-PA*, *PAI-1* polymorphisms and TLE.

First data linking t-PA to epilepsy were provided by Qian [34] who has shown increased *t-PA* mRNA expression in the rat cortex and hippocampus following by pentylenetetrazol (PTZ) inducement. Then, the increase not only in *t-PA* mRNA expression but also in t-PA enzymatic activity has been observed in subsequent mice models [35]. Involvement of t-PA in seizure induction has been demonstrated for the first time by Tsirka who indicated that seizures should be induced by higher injection of either KA or PTZ in t-PA deficiency mice compared with controls [34]. In addition, *t-PA* knock-out mice did not induce seizures even though injecting highest dose of kainic acid (KA) [27], which indicated that the expression of *t-PA* may be associated with the susceptibility of epilepsy. Fillla [36] and Hagen [37] reported that mutation in neuroserpin had been associated with neurological disorders characterized by the presence of seizures, and the anti-epileptic function of neuroserpin had been proved by the fact that application of neuroserpin can diminish progression of seizures during SE induced by KA injection to the amygdala. As a form of neuroserpin, increased expression of *PAI-1* mRNA has also been observed in human TLE with hippocampal sclerosis and focal cortical dysplasia. Previous studies have reported that t-PA and PAI-1 levels can be affected by their gene polymorphisms [38, 39], and thus we speculated that *t-PA* and *PAI-1* gene polymorphisms may be an associated with the occurrence of TLE.

In this context, we adopted a case-control study to investigate the association between the SNPs in *t-PA* (rs2020918, rs4646972) and *PAI-1* (rs1799768) and susceptibility to TLE in Han Chinese population in terms of genetic perspective. Our study of 121 TLE patients and 146 controls found significant differences in genotypic and allelic frequencies of polymorphic sites (rs2020918, rs4646972) between TLE patients and control groups. However, the significant difference of the rs2020918 results was not confirmed after Bonferroni adjustment. To further study the association between *t-PA* polymorphism and the clinical phenotypes, for rs2020918, the TLE patients were divided into two groups according to whether they carried T allele or not (CC and CT+ TT groups). The results showed an obvious difference between cases and controls, which indicated that genotypes carrying T (mutant allele) may be more susceptible to TLE compared with wild-genotype. Moreover, the T allele was higher in TLE patients than that in normal controls, which indicated that T allele may play a risky role in the development of TLE. Likewise, for rs4646972, when subdividing these samples into 311 bp/311 bp and 311 bp/− + −/− groups, we also observed an obvious difference between cases and controls, which indicated that genotypes with 311 bp deletion carriers seem to be more susceptible to TLE compared with wild-genotype. Furthermore, a statistical difference in 311 bp deletion allele was found between TLE patients and normal controls and 311 bp deletion allele may be a risky factor to the occurrence of TLE according to the value of OR.

However, we failed to find a significant difference in genotypic and allelic frequencies of polymorphic site *PAI-1* rs1799768 between TLE and control groups, which suggested that rs1799768 polymorphism may not be associated with the pathogenesis and progression of TLE. The negative results can be explained by many reasons. Firstly, TLE is a complex polygenetic hereditary disease which can be influenced by environmental factors rather than several genetic defects only. Moreover, our results may be affected by regional and racial biases to some extent. One limitation of our study is that most subjects are from Hunan province. Additionally, the effects of environmental risk factors and comorbidity are not taken into consideration as we only investigated one SNP of *PAI-1.* To ensure the replicability of our finding, the independent group of patients should be included in further study.

## Conclusion

In conclusion, the obtained results suggested an apparent association between the *t-PA* polymorphisms and increased TLE susceptibility in the analyzed Chinese Han population, which can enrich the understanding of sporadic TLE susceptibility genes. In order to provide a molecular genetic evidence for clinically seizure risk predication and individual treatment, larger-scale functional and genetic studies investigating different genes are necessary to be carried out in patients from multiple regions to discover new TLE susceptibility genes and gain further insights into its pathogenesis.

## Abbreviations

ApoE: ApolipoproteinE
CNS: Central nervous system
GABBR1: γ-Aminobutyric acid B receptor 1
IL-1 RA: Interleukin-1 receptor antibody
IL-1β: Interleukin-1β
KA: Kainic acid
PAI-1: Plasminogen activator inhibitor 1
PCR-LDR: Polymerase chain reaction-ligase detection reaction
PRNP: Prion protein
PTZ: Pentylenetetrazol
PYDN: Prodynorphin
TLE: Temporal lobe epilepsy
t-PA: Tissue plasminogen activator
